# Forensic Sciences Research: from dream to reality

**DOI:** 10.1080/20961790.2017.1292990

**Published:** 2017-03-16

**Authors:** Duarte Nuno Vieira, Min Shen

**Affiliations:** Faculty of Medicine, University of Coimbra, Coimbra, Portugal; Shanghai Key Laboratory of Forensic Medicine, Shanghai Forensic Service Platform, Institute of Forensic Science, Ministry of Justice, PRC, Shanghai, China

It is with great satisfaction and joy that we present the second issue of *Forensic Sciences Research* (*FSR*). The launching of a new journal is always a complex process, as it is essential to obtain trust and credibility within the scientific community, and especially in the field in question. This second issue shows that we have achieved this objective.

*FSR* will be attentive to the winds of change, the phenomena of the present, the relentless pace of technical and scientific development, constant social and cultural change, legal needs, and the needs, anxieties and demands of justice and the forensic science community. This journal aims to contribute to modern and dynamic forensic services – services that pulsate with the rhythm of life and social needs, services able to provide highly qualified forensic expertise; services that are welcoming and inspire confidence; services in which all efforts are made to relieve the anguish and suffering of victims and families; services that fulfil their mission with competence, quality, independence, impartiality, objectivity, truthfulness, diligence and prudence.

We want to publish papers that are motivating and creative, written by top professionals and using the most recent and sophisticated technology. But we also want to be an open door for disseminating the reality, anguish and thoughts of those who provide forensic services in particularly difficult circumstances and environments. That is the challenge that has been set. That is the challenge that we will meet.

The international editorial board of *FSR* comprises experts distinguished not only by their professionalism, but also their enthusiasm, determination, dedication, availability and spirit of solidarity with those that come to them. Experts who are aware of the need to constantly update their technical and scientific knowledge, to exchange experiences, establish partnerships nationally and abroad, share reflections and concerns, and analyse results critically. Experts who are able to break with routine and do away with passivity, inertia and lack of ambition. A team characterized by its energy, initiative, creativity, imagination and daring. A team that attempts daily to enforce the values and causes in which we believe, continuously nourishing its ability to supply ever better forensic services, and its capacity to build. A team that pursues these goals with enthusiasm and hope, knowing that it is our duty to do what is difficult, without capitulating to easy solutions.

This second volume represents the leap of hope necessary to propel *FSR* to where dreams and reality meet.

This is a road that we will walk together. Through the research published in *FSR*, we will continue to fight for the causes and values of the forensic services we provide, and for the continued improvement of our methods, equipment, facilities and staff. We will continue to implement a project both sturdy and exemplary, which assumes its role as standard-bearer of excellence and quality in all forensic sectors, without compromise. This project will continue to affirm its role as a leading journal with solid roots, one that faces the challenges it encounters and perceives them as goals.

We are fully aware that it is possible to do more and better. And we want to do more and better! We believe in the potential of this journey. We believe that we will always have a wide margin for progress. We believe that it is always possible to invent a future that is even better than the present and is worth fighting for. We believe in keeping our dream alive and making it grow. And above all, we believe that, together, we can do incredible things. We are looking forward to doing that with you.

